# Efficacy and safety of combination PD‐1/PD‐L1 checkpoint inhibitors for malignant solid tumours: A systematic review

**DOI:** 10.1111/jcmm.15991

**Published:** 2020-10-20

**Authors:** Qigu Yao, Lihu Gu, Rong Su, Bangsheng Chen, Hongcui Cao

**Affiliations:** ^1^ State Key Laboratory for Diagnosis and Treatment of Infectious Diseases The First Affiliated Hospital College of Medicine Zhejiang University Hangzhou City China; ^2^ Department of General Surgery HwaMei Hospital University of Chinese Academy of Sciences Ningbo City China; ^3^ Emergency Medical Center The Second Hospital of Yinzhou Ningbo City China; ^4^ Zhejiang Provincial Key Laboratory for Diagnosis and Treatment of Aging and Physic‐chemical Injury Diseases Hangzhou City China

**Keywords:** adverse events, meta‐analysis, PD‐1/PD‐L1 inhibitors, solid tumours

## Abstract

Treatment of multiple malignant solid tumours with programmed death (PD)‐1/PD ligand (PD‐L) 1 inhibitors has been reported. However, the efficacy and immune adverse effects of combination therapies are controversial. This meta‐analysis was performed with PubMed, Web of Science, Medline, EMBASE and Cochrane Library from their inception until January 2020. Random‐effect model was adopted because of relatively high heterogeneity. We also calculated hazard ratio (HR) of progression‐free survival (PFS), overall survival (OS) and risk ratio (RR) of adverse events (AEs), the incidence of grade 3‐5 AEs by tumour subgroup, therapeutic schedules and therapy lines. Nineteen articles were selected using the search strategy for meta‐analysis. Combined PD‐1/PD‐L1 inhibitors prolonged OS and PFS (HR 0.72, *P* < 0.001) and (HR 0.66, *P* < 0.001). In addition, incidence of all‐grade and grade 3‐5 AEs was not significant in the two subgroup analyses (HR 1.01, *P* = 0.31) and (HR 1.10, *P* = 0.07), respectively. Our meta‐analysis indicated that combination therapy with PD‐1/PD‐L1 inhibitors had greater clinical benefits and adverse events were not increased significantly.

## 1 BACKGROUND

In the past 10 years, programmed death (PD)‐1 and PD ligand (PD‐L)1 have become increasingly attractive for therapy of many solid tumours.^1^ PD‐1/PD‐L1 checkpoint inhibitors, such as pembrolizumab, nivolumab and atezolizumab, have been approved by the US Food and Drug Administration for 17 different types of advanced unresectable cancers, in first‐ and later‐line treatment settings.^2^ These agents are key mediators of local immunosuppression in the tumour microenvironment (TME) and regulate T‐cell activation and proliferation to attack tumour cells.^2^, ^3^ PD‐1/PD‐L1 inhibitors have demonstrated clinical efficacy in terms of overall survival (OS) and progression‐free survival (PFS).^4^, ^5^

However, tumour resistance, especially acquired resistance, blocks further, widespread use of PD‐1/PD‐L1 inhibitors. Furthermore, pancreatic and prostate cancers are particularly resistant to this treatment approach.^6^ Therefore, combination strategies have been suggested. They may exert immunopotentiating effects by increasing the mutational load in cancer cells and increasing the sensitivity of tumour cells to T cells.^7^ In non–small‐cell lung cancer (NSCLC), PD‐1/PD‐L1 inhibitors initially demonstrated efficacy as monotherapy.^8^ Combination of platinum‐based chemotherapy with PD‐1/PD‐L1 inhibitors improved efficacy.^4^, ^9^, ^10^, ^11^ The efficacy of combination of PD‐1/PD‐L1 inhibitors with ipilimumab is also encouraging in melanoma.^12^ Besides, combination of PD‐1/PD‐L1 inhibitors with nab‐paclitaxel in breast cancer^13^ and with dabrafenib and trametinib in melanoma^14^ has shown similar efficacy. There are now >100 ongoing clinical trials of PD‐1/PD‐L1 inhibitors as monotherapy or in combination with other agents in different tumour types.^15^ Nevertheless, the use of these agents can be limited by adverse events (AEs), such as nausea, fatigue, decreased appetite, diarrhoea and vomiting.^16^ The clinical benefit associated with combination PD‐1/PD‐L1 inhibitors should be balanced against associated toxicity.

Addition of PD‐1/PD‐L1 inhibitors to treatment remains controversial, and individual studies are not sufficient to clarify this. Whether PD‐1/PD‐L1 checkpoint inhibitors will achieve significant efficacy for all tumour types or different therapeutic schedules is still up for question. Therefore, we performed a meta‐analysis of phase II/III randomized controlled trials to compare the efficacy and safety of combination PD‐1/PD‐L1 checkpoint inhibitors for malignant solid tumours. It is important for clinical policymakers to explore the degree of efficacy in different tumour types, therapeutic schedules and therapy lines. Additionally, the incidence of AEs may provide clinicians with important and clinically useful information.

## 2 MATERIALS AND METHODS

### 2.1 Search strategy

This meta‐analysis was performed with PubMed, Web of Science, Medline, EMBASE and Cochrane Library from their inception until January 2020 to identify relevant studies. A combination of free‐text terms and medical subject headings terms was used for the subject search. Search terms included “nivolumab” OR “BMS 936558” OR “BMS 936559” OR “MDX 1105” OR “pembrolizumab” OR “lambrolizumab” OR “MK 3475” OR “pidilizumab” OR “CT 011” OR “durvalumab” OR “MEDI 4736” OR “atezolizumab” OR “MPDL 3280a” OR “avelumab” OR “AMP 224” OR “PD‐1” OR “PD‐L1” OR “programmed death 1” OR “programmed death ligand 1” OR “programmed cell death ligand 1” OR “programmed death ligand 1” OR “B7‐H1” OR “CD274” AND “tumor” OR “cancer” OR “carcinoma” OR “neoplasm” OR “malignancy” OR “sarcoma”. We also had two researchers independently screen the titles and abstracts of the retrieved articles.

### 2.2 Study selection

Studies were included if they met the following criteria. (a) Literature type: phase II/III randomized controlled trials. (b) The experimental intervention group was treated with combination PD‐1/PD‐L1 checkpoint inhibitors with other therapies (immunotherapy, chemotherapy, targeted therapy and radiotherapy), whereas the control group received other therapies without PD‐1/PD‐L1 inhibitors. (c) Efficacy and safety data were available. Exclusion criteria were as follows: (a) studies with post‐operative adjuvant therapy and neoadjuvant therapy; (b) not in English; and (c) multiple articles that analysed the same trials. In the latter case, we analysed the latest data.

### 2.3 Data extraction and quality assessment

Data from each study were extracted by two researchers independently. A third researcher was consulted to reach a majority decision. The following information was used: (a) authors' names, year of publication, tumour type, therapy lines, sample size and interventions; and (b) the primary efficacy outcomes were OS and PFS, and the secondary outcome was AEs. The meta‐analysis was conducted in accordance with the guidelines of the Preferred Reporting Items for Systematic Review and Meta‐Analysis Protocols (PRISMA‐P) 2015 statement.^17^

### 2.4 Statistical analysis

We calculated the hazard ratio (HR) and 95% confidence interval (CI) for OS and PFS and the risk ratio (RR) and 95% CI for AEs. We also performed subgroup analyses of OS, PFS and incidence of grade 3‐5 AEs for patients with different tumour types, therapeutic schedules and therapy lines. Revman version 5.3 (The Cochrane Collaboration) was used to perform the meta‐analysis. Heterogeneity between studies was evaluated using the chi‐squared test and *I* ^2^ statistics. Because of the complexity of the control conditions and the variety of solid tumours, a random‐effect model was used to enhance the credibility of the results. We used Begg's and Egger's tests with Stata SE version 12 (Stata Corporation), with significance set at *P* < 0.1, to evaluate publication bias. All the statistical tests were two‐sided, and *P* < 0.05 was considered statistically significant.

## 3 RESULTS

### 3.1 Eligible studies and characteristics

**Figure 1:**
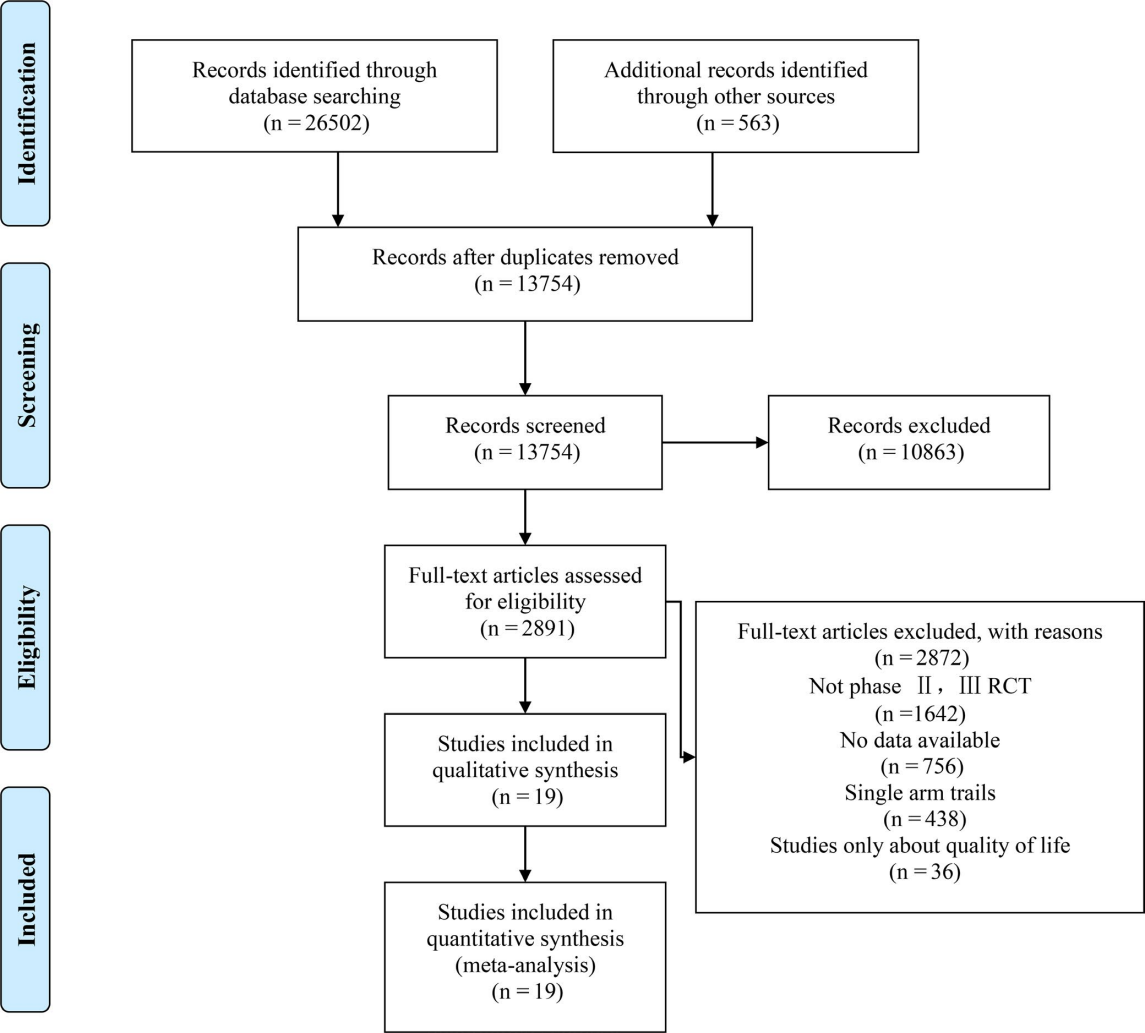
Flow chart of study selection

The search strategy generated 26 502 relevant clinical records from the five databases. After screening and eligibility assessment, 19 eligible^5^, ^34^ phase II/III randomized controlled trials were selected for meta‐analysis, including 10 178 patients. The detailed search and study selection process is shown in Figure 1. In addition, RCTs was evaluated with the Cochrane Collaboration tool, which demonstrated relatively high methodological quality (Figures S1 and S2).

### 3.2 Study characteristics and quality

The basic characteristics of the selected studies are shown in Table 1. Most of the 19 studies were of chemotherapy and targeted therapy, including 8 chemotherapy + PD‐1/PD‐L1 vs chemotherapy; 6 targeted therapy + PD‐1/PD‐L1 vs. targeted therapy; 2 immunotherapy (ipilimumab) + PD‐1/PD‐L1 vs immunotherapy (ipilimumab); 1 best supportive care (BSC) + PD‐1/PD‐L1 vs BSC; 1 chemotherapy + targeted therapy + PD‐1/PD‐L1 vs chemotherapy + targeted therapy; and 1 chemoradiotherapy + PD‐1/PD‐L1 vs chemoradiotherapy. To analyse comparability further, we also recorded the basic tumour types and the different lines of therapy. There were 7 different tumour types, namely NSCLC (n = 6), melanoma (n = 3), renal carcinoma (n = 4), SCLC (n = 2), hepatocellular carcinoma (n = 1), colorectal cancer (n = 1), breast cancer (n = 1) and head and neck carcinoma (n = 1). There were 14 trials with first‐line therapy and 5 with second or beyond lines of therapy.

**Table 1:** Study characteristics

Author, year	Phase	Tumour	Line	Sample size		Interventions	
						Experimental	Control
Antonia 2018	III	NSCLC	1L	476	237	Chemoradiotherapy + Durvalumab	Chemoradiotherapy + Placebo
Ascierto 2019	II	Melanoma	1L	60	60	Dabrafenib + Trametinib + Pembrolizumab	Dabrafenib + Trametinib + Placebo
Borghaei 2019	IIIB/IV	NSCLC	1L	60	63	Pemetrexed‐carboplatin + Pembrolizumab	Pemetrexed‐carboplatin
Eng 2019	III	Colorectal cancer	2L	183	90	Cobimetinib + Atezolizumab	Regorafenib
Ferris 2016	III	Carcinoma of the Head and Neck	2L	240	121	Chemotherapy + Nivolumab	Chemotherapy
Finn 2020	III	HCC	2L	278	135	BSC + Pembrolizumab	BSC + Placebo
Gandhi 2018	II	NSCLC	1L	410	206	Pemetrexed + Platinum‐based drug + Pembrolizumab	Pemetrexed + Platinum‐based drug + Placebo
Hodi 2016	II	Melanoma	1L	95	47	Ipilimumab + Nivolumab	Ipilimumab + Placebo
Hodi 2018	III	Melanoma	1L	314	315	Ipilimumab + Nivolumab	Ipilimumab
Horn 2018	III	SCLC	2L	201	202	Chemotherapy + Atezolizumab	Chemotherapy + Placebo
McDermott 2018	II	RCC	1L	101	101	Bevacizumab + Atezolizumab	Sunitinib
Motzer 2019	III	RCC	1L	442	444	Axitinib + Avelumab	Sunitinib
Paz‑Ares 2018	III	NSCLC	1L	278	281	Chemotherapy + Pembrolizumab	Chemotherapy + Placebo
Paz‐Ares 2019	III	SCLC	2L	268	269	Platinum–etoposide + Durvalumab	Platinum–etoposide
Reck 2019	III	NSCLC	1L	400	400	Bevacizumab + Chemotherapy + Paclitaxel + Atezolizumab	Bevacizumab + Chemotherapy + Paclitaxel
Rini (1) 2019^a^	III	RCC	1L	454	461	Bevacizumab + Atezolizumab	Sunitinib
Rini (2) 2019^a^	III	RCC	1L	432	429	Axitinib + Pembrolizumab	Sunitinib
Schmid 2020	III	Breast Cancer	1L	451	451	Nab‐paclitaxel + Atezolizumab	Nab‐paclitaxel + Placebo
West 2019	III	NSCLC	1L	483	240	Chemotherapy + Atezolizumab	Chemotherapy

Abbreviations: 1L, first line; 2L, second line or beyond; BSC, best supportive care; HCC, hepatocellular carcinoma; NSCLC, non–small‐cell lung cancer; RCC, renal cell carcinoma; SCLC, small‐cell lung cancer.

^a^ Rini published two articles in the same year. We marked Rini (1) and Rini (2) in order to make a better distinction.

### 3.3 OS

**Figure 2:**
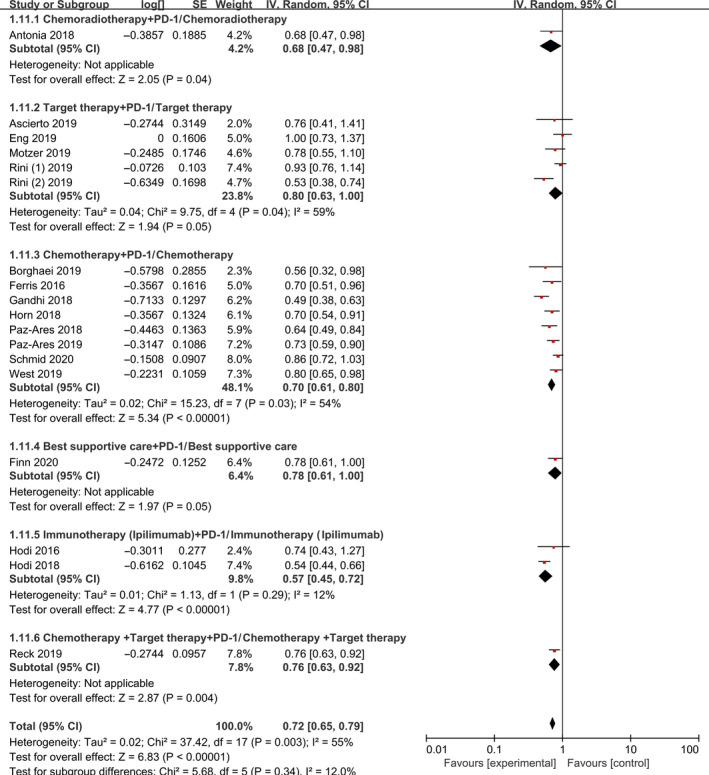
Forest Plot of Hazard ratio of OS based on therapeutic schedules in total population

**Figure 3:**
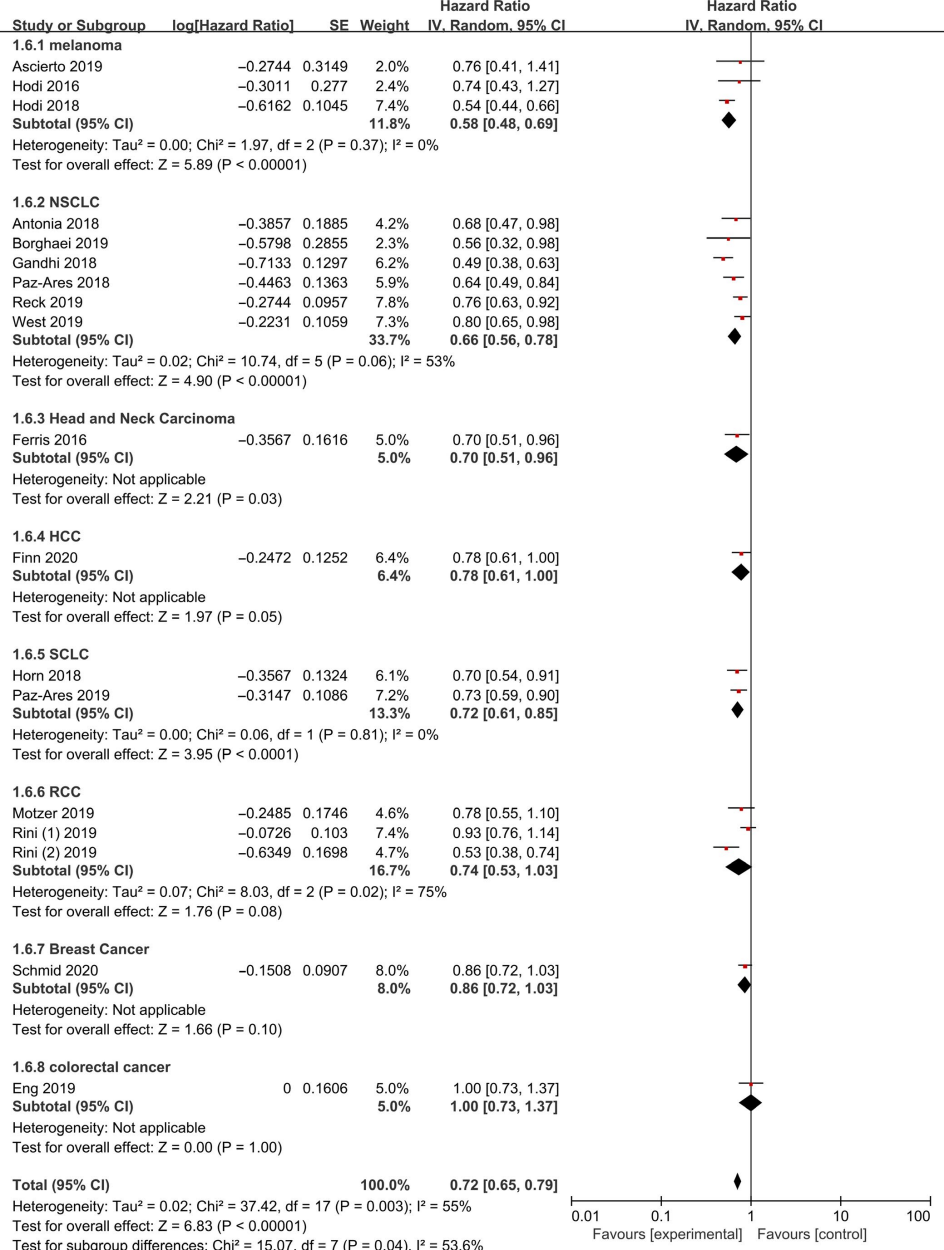
Forest Plot of Hazard ratio of OS based on tumour types in total population

OS was reported in 18 studies. Subgroup analyses for OS are summarized in Figure 2. According to the different therapeutic schedules, tumours and therapy lines, we conducted three subgroup analyses. Combined PD‐1/PD‐L1 inhibitors prolonged OS [HR 0.72, 95% CI (0.65‐0.79), *P* < 0.001]. Eighteen of the selected studies examined HR of OS based on therapeutic schedules and tumour types in total population (Figures 2 and 3). PD‐1/PD‐L1 inhibitors combined with chemotherapy (*P* < 0.0001), targeted therapy (*P* = 0.05), immunotherapy (ipilimumab) (*P* < 0.001) and chemoradiotherapy (*P* = 0.004) was associated with better OS compared with the control groups. Immunotherapy (ipilimumab) plus PD‐1/PD‐L1 had the greatest effect on OS [HR 0.57, 95% CI (0.45‐0.72), *P* < 0.001]. OS was significantly improved in melanoma (*P* < 0.001), NSCLC (*P* < 0.001) and SCLC (*P* < 0.001), and melanoma and NSCLC had significantly better clinic benefit (HR 0.58, *P* < 0.001) and (HR 0.66, *P* < 0.001), respectively. Combination therapy with PD‐1/PD‐L1 inhibitors significantly prolonged OS after first‐line treatment [HR 0.69, 95% CI (0.61‐0.79), *P* < 0.001] and second or additional lines of treatment [HR 0.76, 95% CI (0.68‐0.86), *P* < 0.001]. In addition, combination first‐line treatment with PD‐1/PD‐L1 inhibitors had better clinical efficacy than second or additional lines of therapy (Figure S3).

### 3.4 PFS

**Figure 4:**
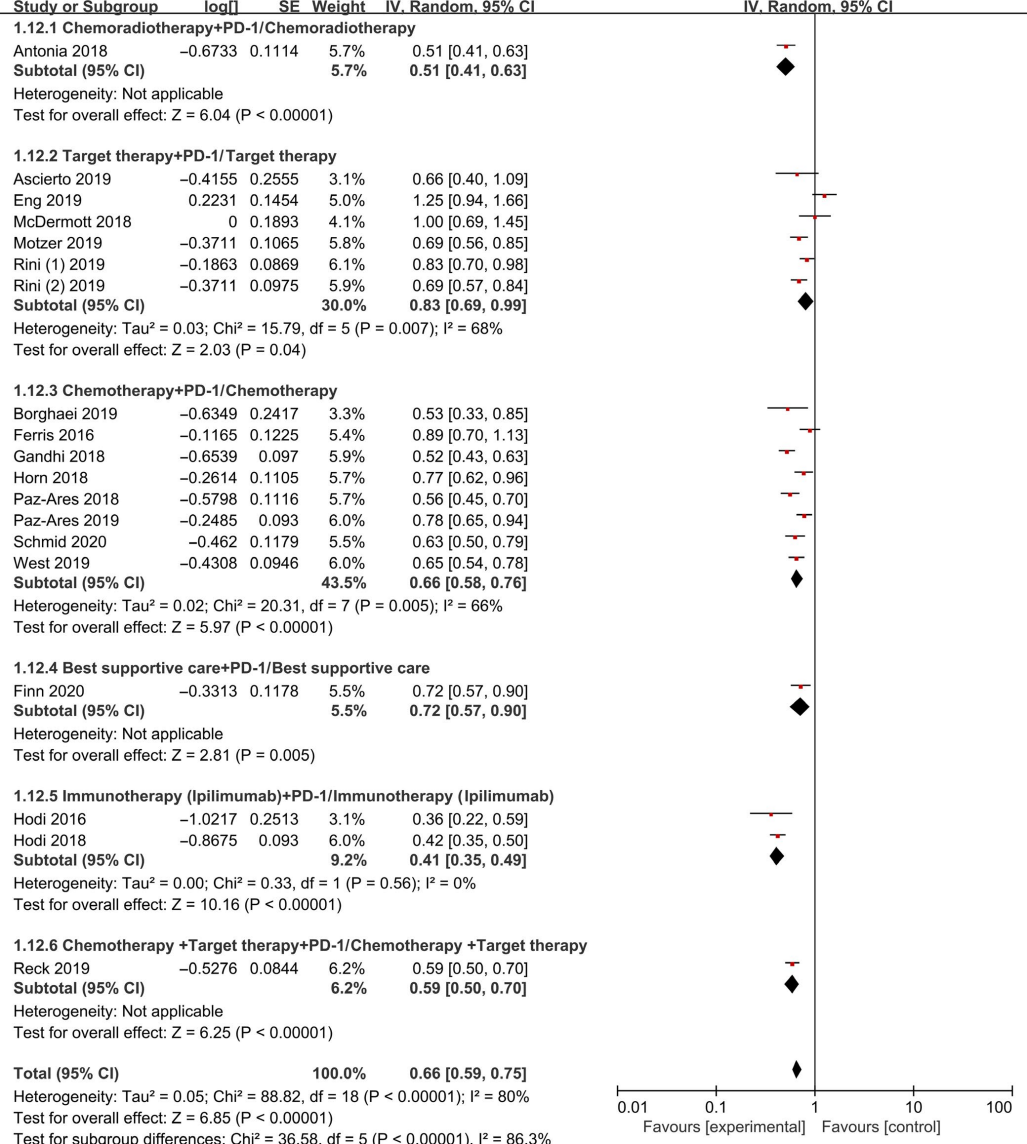
Forest Plot of Hazard ratio of PFS based on therapeutic schedules in total population

**Figure 5:**
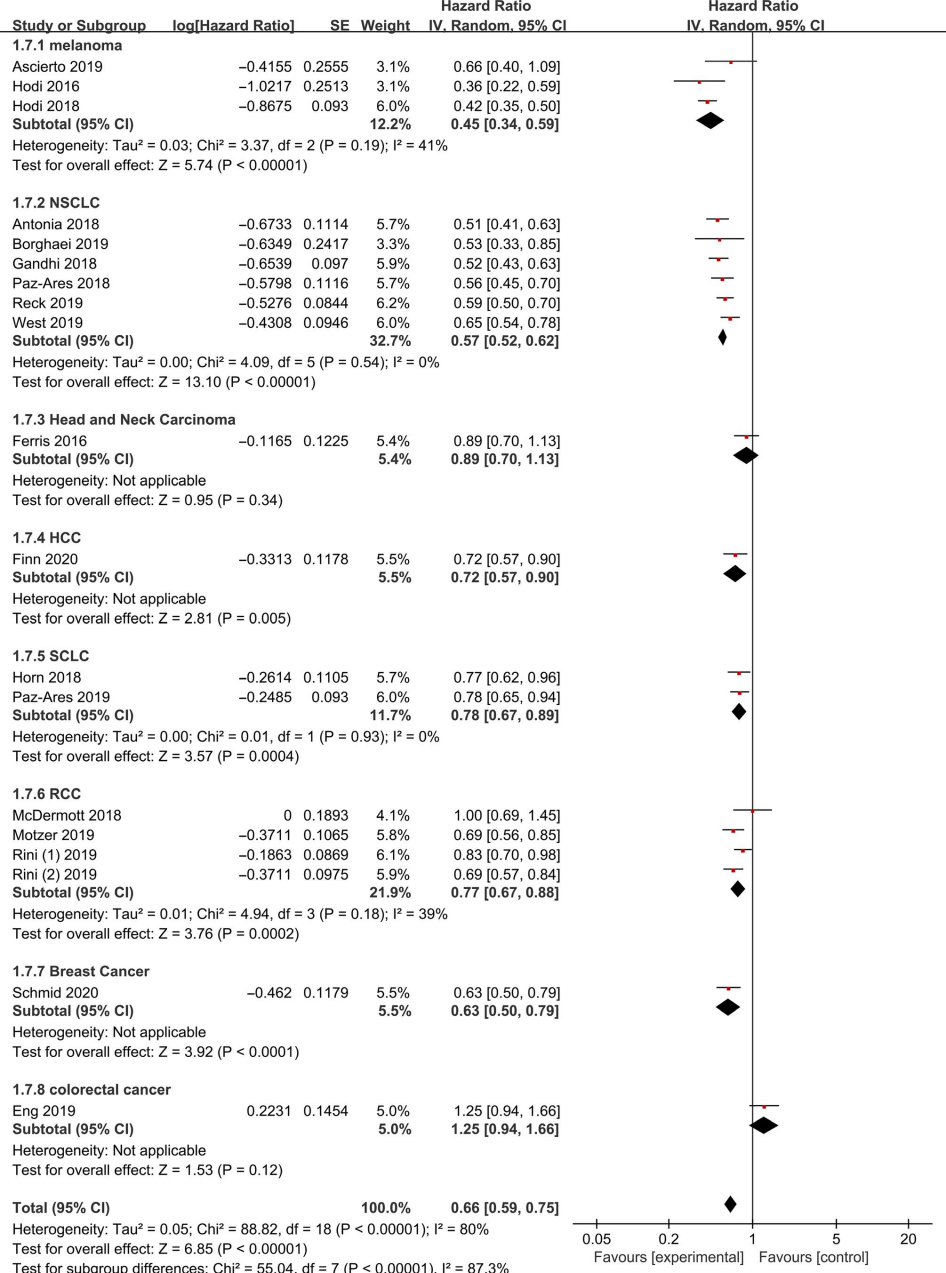
Forest Plot of Hazard ratio of PFS based on tumour types in total population

PFS was reported in all 19 studies. Results of subgroup analyses for PFS are summarized in Figure 4. We conducted three subgroup analyses according to different of therapeutic schedules, tumours and therapy lines. Combination immunotherapy significantly prolonged PFS [HR 0.66, 95% CI 0.59‐0.75, *P* < 0.001]. When the 19 studies were grouped by therapeutic schedules or tumour types, our meta‐analysis showed that all groups achieved different degrees of benefit (Figures 4 and 5). Immunotherapy (ipilimumab) plus PD‐1/PD‐L1 inhibitor had the most significant effect [HR 0.41, 95% CI (0.35‐0.49), *P* < 0.001]. Among the tumour types, melanoma showed the greatest benefit [HR 0.45, 95% CI (0.34‐0.59), *P* < 0.001]. We demonstrated that combination therapy with PD‐1/PD‐L1 inhibitors had longer PFS in first‐line than in second or additional lines of therapy [HR 0.59, 95% CI (0.52‐0.66), *P* < 0.001] and [HR 0.85, 95% CI (0.73‐1.00), *P* = 0.06] (Figure S4).

### 3.5 Incidence of grade 3‐5 AEs

**Figure 6:**
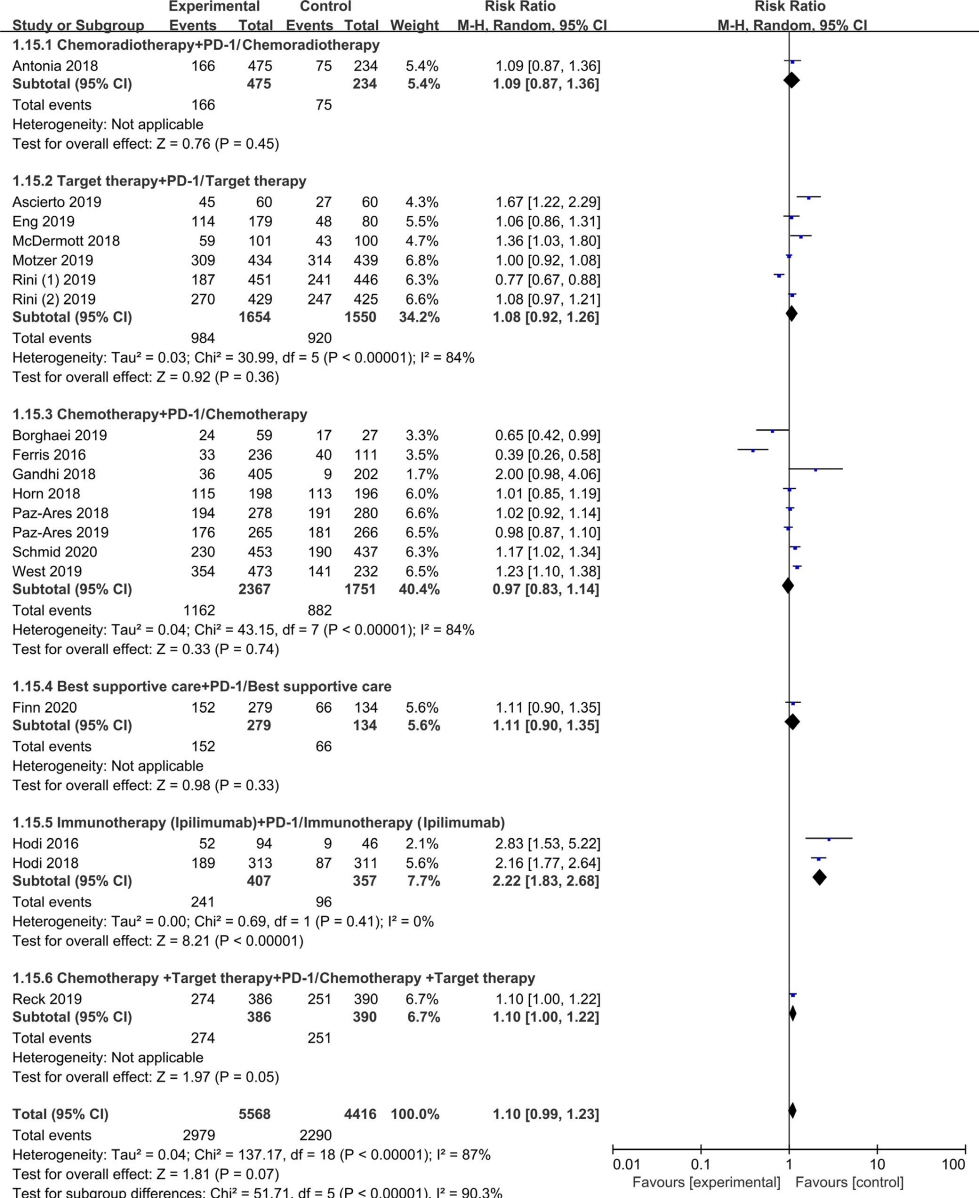
Forest Plot of Hazard ratio of grade 3‐5 AEs rates based on therapeutic schedules in total population

The incidence of grade 3‐5 AEs was examined in 5568 patients in the experimental groups and 4416 patients in the control groups. We performed subgroup analysis according to the different therapeutic schedules and tumour types. The incidence of grade 3‐5 AEs was not significant in the 2 subgroup analyses (HR 1.10, 95% CI 0.99‐1.23, *P* = 0.07). According to the subgroup analysis, immunotherapy (ipilimumab) plus PD‐1/PD‐L1 inhibitor had AEs [HR 2.22, 95% CI (1.83‐2.68), *P* < 0.001], compared with the control group (Figure 6 and Figure S5).

### 3.6 Incidence of all‐grade and grade 3/4 AEs

The incidence of all‐grade and grade 3/4 AEs was examined in 5315 patients in the experimental groups and 4258 patients in the control groups. A total of 9573 patients experienced AEs of any grade. Combination therapy with PD‐1/PD‐L1 inhibitors had no significant advantage [RR 1.01, 95% CI (0.99‐1.01), *P* = 0.31] compared with the control group (Figure S6). Due to the large number of AEs reported, we selected the most common all‐grade and grade 3/4 AEs for analysis (Table 2). The most common all‐grade AEs were fatigue (RR = 0.99), nausea (RR = 0.97), diarrhoea (RR = 1.08) and decreased appetite (RR = 0.98). The incidence of most AEs was not increased by PD‐1/PD‐L1 inhibitors, except for a significant decrease in anaemia (all‐grade RR 0.70, *P* = 0.003, grade 3/4 RR 0.71, *P* = 0.04) and significant increase in rash (all‐grade RR 1.46, *P* < 0.0001, grade 3/4 RR 1.08, *P* < 0.0001).

**Table 2:** Subgroup analysis of the adverse events (AEs)

Experimental vs. control	No. of studies	RR	95% CI	*P*	Heterogeneity (*I* ^2^)
Any grade adverse events	19	1.01	0.99‐1.02	.31	68
Any grade fatigue	19	0.99	0.91‐1.07	.79	48
Any grade nausea	19	0.97	0.83‐1.13	.84	84
Any grade diarrhoea	19	1.08	0.90‐1.29	.42	87
Any grade decreased appetite	19	0.98	0.84‐1.15	.79	72
Any grade vomiting	17	1.05	0.83‐1.33	.67	79
Any grade anaemia	15	0.70	0.56‐0.88	.003	89
Any grade rash	14	1.46	1.28‐1.66	<.0001	21
Any grade constipation	13	1.08	0.98‐1.19	.13	0
Any grade asthenia	13	0.92	0.82‐1.03	.15	8
3/4 grade adverse events	19	1.08	1.04‐1.12	<.0001	86
3/4 grade nausea	19	1.06	0.74‐1.52	.76	0
3/4 grade fatigue	19	0.94	0.66‐1.35	.76	49
3/4 grade decreased appetite	19	1.26	0.76‐2.08	.37	27
3/4 grade diarrhoea	19	1.25	0.92‐1.68	.15	34
3/4 grade vomiting	16	0.91	0.58‐1.41	.66	0
3/4 grade anaemia	15	0.71	0.51‐0.99	.04	75
3/4 grade rash	15	1.61	0.95‐2.73	.08	0
3/4 grade asthenia	13	0.87	0.61‐1.25	.46	4
3/4 grade constipation	13	1.63	0.70‐3.77	.26	0

### 3.7 Publication bias

Begg's test (*P = *0.198 > 0.1) and Egger's test (*P = 0*.34 > 0.1) showed no significant publication bias in OS (Figure S7).

## 4 DISCUSSION

In the past 10 years, >10 cancers have been recommended for treatment with PD‐1/PD‐L1 checkpoint inhibitors, with objective response rates of 10%‐30% and good toxicity.^35^ Compared with traditional therapies, PD‐1/PD‐L1 inhibitors can prolong survival because of the memory of the adaptive immune system.^36^ Nevertheless, we have to acknowledge that many patients do not benefit from the treatment or relapse after a period of response, especially in breast and colon cancers.^20^, ^33^, ^37^ Tumour‐mediated mechanisms of immunotherapy resistance are improved by synergism with targeted therapies or chemotherapy.^38^ Many studies have demonstrated that combination with chemotherapy, molecular‐targeted therapy and immunotherapy has good curative effect and adequate safety.^5^, ^22^, ^39^

In the presence of efficacy based on therapeutic schedules, we found that adding PD‐1/PD‐L1 inhibitors to various therapeutic schedules achieved different degrees of clinical benefit. In 8 chemotherapy groups, combined chemotherapy with PD‐1/PD‐L1 inhibitors achieved the impressive efficacy, which was consistent with recent clinical trials.^19^, ^33^ A pre‐clinical trial^40^ showed that chemotherapy induces PD‐L1 overexpression via nuclear factor‐κB, which aggravates immunosuppression in ovarian cancer. The mechanisms of action of chemotherapeutic agents include the death of tumour cells with immunogenicity, reduced immunosuppressive effect and sensitization of tumour cells to immune effector cells.^40^ When it comes to adding PD‐1/PD‐L1 inhibitors, many studies have investigated the mechanism. Firstly, combination therapies can increase cross‐presentation of tumour antigens and up‐regulation of major histocompatibility complex (MHC) class I antigens.^41^ Secondly, in the presence of interleukin (IL)‐2, IL‐5 and other cytokines, combination therapies enhance CD8 T‐cell activation and their ability to attack tumour cells.^42^

Our research indicated that the addition of PD‐1/PD‐L1 inhibitors prolonged OS and PFS notably in molecular‐targeted treatment. There has been an increase in the use of anti‐vascular endothelial growth factor (VEGF) agents for molecular‐targeted therapy.^43^ VEGF, IL‐10 and prostaglandin E2 are released by cells and exert systemic immunosuppressive effects in the TME.^44^ Consequently, these cytokines and growth factors may down‐regulate anticancer immunity of cytotoxic T lymphocytes.^45^ Anti‐VEGF agents have been shown to have multiple mechanisms of action.^43^, ^46^ Some studies^47^, ^48^ have reported that anti‐VEGF agents up‐regulate PD‐L1 on endothelial cells and tumour cells and cause abnormal vascularization in mouse models, which aggravates immunosuppression. It has been suggested that treatment with PD‐1/PD‐L1 inhibitors ameliorates immune escape and promotes normalization of tumour vasculature.^44^, ^49^

Only one included article mentioned that combined PD‐1/PD‐L1 inhibitors with radiotherapy improved the curative effect. When radiotherapy is combined with PD‐1/PD‐L1 inhibitors, it can increase inflammatory processes, restrain leucocyte adhesion to ECs, promote apoptosis and reduce oxidative burst in macrophages.^50^ In NSCLC, radiotherapy can up‐regulate tumour cell PD‐L1 expression.^51^

Besides, the greatest benefit was observed with immunotherapy (ipilimumab) when plus PD‐1/PD‐L1 inhibitors for malignant solid tumours. Combination of PD‐1 and cytotoxic T lymphocyte‐associated antigen‐4 has the potential to increase response rates in patients with renal cell carcinoma.^52^ Other immune checkpoints, including lymphocyte activation gene 3 and T‐cell immunoglobulin 3, may also enhances antitumour T‐cell immunity when PD‐1/PD‐L1 inhibitors are added.^53^

In subgroup analysis based on tumour types, our meta‐analysis demonstrated that OS and PFS were increased in melanoma more than in other tumours. Just as Sharma^37^ said, melanoma had substantial effect on immunological activity and potential synergy when combination strategy was designed with molecularly targeted therapy. Some studies have demonstrated that *BRAF*‐targeted therapy increases expression of antigenic proteins, restores MHC‐I surface expression, increases T‐cell infiltration, facilitates T‐cell cytotoxicity and a more favourable TME,^54^, ^55^ which helps PD‐1/PD‐L1 checkpoint inhibitors to reduce the effect of immune resistance.^56^ The OS and PFS of first‐line treatment were significantly higher than those of second‐line or beyond treatment.

Our meta‐analysis demonstrated that combination treatment with PD‐1/PD‐L1 checkpoint inhibitors did not significantly increase incidence of all‐grade AEs. Nearly 95% of patients experienced at least 1 AE, which is consistent with Hoffner.^16^ Second, when immunotherapy (ipilimumab) plus PD‐1/PD‐L1 or combination PD‐1/PD‐L1 inhibitors used in melanoma, the rate of grade 3‐5AEs showed AEs increased significantly, which is also consistent with previous results.^57^, ^58^ The most common AEs were fatigue, nausea and diarrhoea. The incidence of rash was raised rapidly, which might be attributed to the use of PD‐1/PD‐L1 inhibitors. It has been shown that PD‐1 blockade increases the risk of immune‐mediated AEs when combined with chemotherapy.^59^ We think that the decline of anaemia could be due to the addition of PD‐1/PD‐L1 inhibitors 3267 patients of included studies receiving lower dose chemotherapy in experiment group than 2470 patients in control group.^5^, ^19^, ^21^, ^23^, ^26^, ^29^, ^33^, ^34^

As far as we known, the present study is the first to analyse comprehensively the efficacy and safety of combination treatment with PD‐1/PD‐L1 checkpoint inhibitors for malignant solid tumours. Our study had several advantages. First, the data were extracted from 19 multicenter phase II/III randomized controlled trials that involved over 10 000 patients, which had high‐quality designs. Second, multiple subgroups were analysed, according to the types of tumours, agents and therapies. Third, we evaluated the incidence of all‐grade AEs and grade 3‐5 AEs, respectively.

Our study also had some limitations. First, some of the included subgroups were too small to evaluate effectively, such as HCC, breast cancer, colorectal cancer and chemoradiotherapy. Second, the promising biomarkers of PD‐L1 tumour proportion scores and tumour mutation burden were not measured in subgroup analysis because of the lack of sufficient data. Third, we did not consider drug doses, or baseline patient characteristics, such as sex and age.

The clinic benefits and risk of AEs, as well as costs, should be considered. Our findings revealed the efficacy of combination treatment with PD‐1/PD‐L1 checkpoint inhibitors for malignant solid tumours, and it did not result in unexpected toxicity. In the future, detection of PD‐L1 expression, microsatellite analysis and combination with other therapies, such as molecular‐targeted agent, chemotherapy or radiotherapy, will allow further subgroup validation in order to select the most appropriate and economic treatment.

## 5 CONCLUSIONS

For malignant solid tumours, patients treated with first‐ or second‐line combination therapy with PD‐1/PD‐L1 inhibitors had significantly prolonged PFS and OS, with only a small increase in the incidence of AEs.

## CONFLICT OF INTEREST

The authors declare that they have no conflict of interest.

## AUTHOR CONTRIBUTIONS

**Qigu Yao:** Data curation (lead); formal analysis (lead). **Lihu Gu:** Data curation (equal); formal analysis (equal); methodology (equal); software (equal). **Rong Su:** Formal analysis (equal); methodology (equal); software (equal). **Bangsheng Chen:** Data curation (equal); formal analysis (equal); methodology (equal); software (equal); supervision (equal). **Hongcui Cao:** conceptualization (lead); supervision (lead); writing‐review and editing (lead).

## ETHICAL APPROVAL

This article does not contain any studies with human participants or animals performed by any of the authors.

## Supporting information

Figures S1‐S7

## Data Availability

The data used to support the findings of this study are included within the article.
