# Supplementary material for: Efficacy and safety of combination PD‐1/PD‐L1 checkpoint inhibitors for malignant solid tumours: A systematic review
Source: J Cell Mol Med. 2020 Oct 20;24(22):13494–506. doi: 10.1111/jcmm.15991 (PMC7701512; doi:10.1111/jcmm.15991)
Supplement: Supplementary file 1 — Figures S1‐S7 [file JCMM-24-13494-s001.docx]

**Supplementary data**

**Efficacy and safety of combination** **PD-1/PD-L1 checkpoint inhibitors for malignant solid tumors: a systematic review**

Qigu Yao^1^ **^†^**, Lihu Gu^2^ **^†^**, Rong Su^1^, Bangsheng Chen^3^, Hongcui Cao^1,4^*

1 State Key Laboratory for Diagnosis and Treatment of Infectious Diseases, The First Affiliated Hospital, College of Medicine, Zhejiang University, 79 Qingchun Rd., Hangzhou City 310003, China

2 Department of General Surgery, HwaMei Hospital, University of Chinese Academy of Sciences, Ningbo, Zhejiang, China

3 Emergency Medical Center, the Second Hospital of Yinzhou, 998 North Qianhe Road, Yinzhou District, Ningbo, 315100, Zhejiang, China

4 Zhejiang Provincial Key Laboratory for Diagnosis and Treatment of Aging and Physic-chemical Injury Diseases, 79 Qingchun Rd, Hangzhou 310003, China

**^†^** Qigu Yao and Lihu Gu contributed equally.

*Corresponding author:

Hongcui Cao, State Key Laboratory for Diagnosis and Treatment of Infectious Diseases, The First Affiliated Hospital, College of Medicine, Zhejiang University, 79 Qingchun Rd., Hangzhou City 310003, China. Tel: 86-571-87236451; Fax: 86-571-87236459

E-mail: hccao@zju.edu.cn


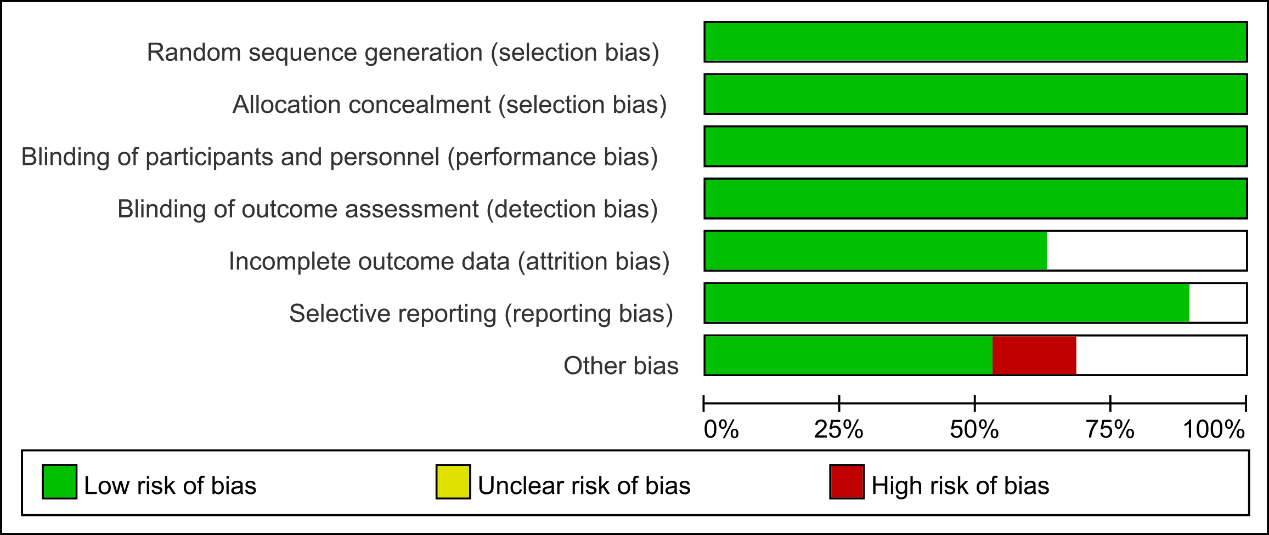


Supplemental Fig. S1 Risk of bias graph


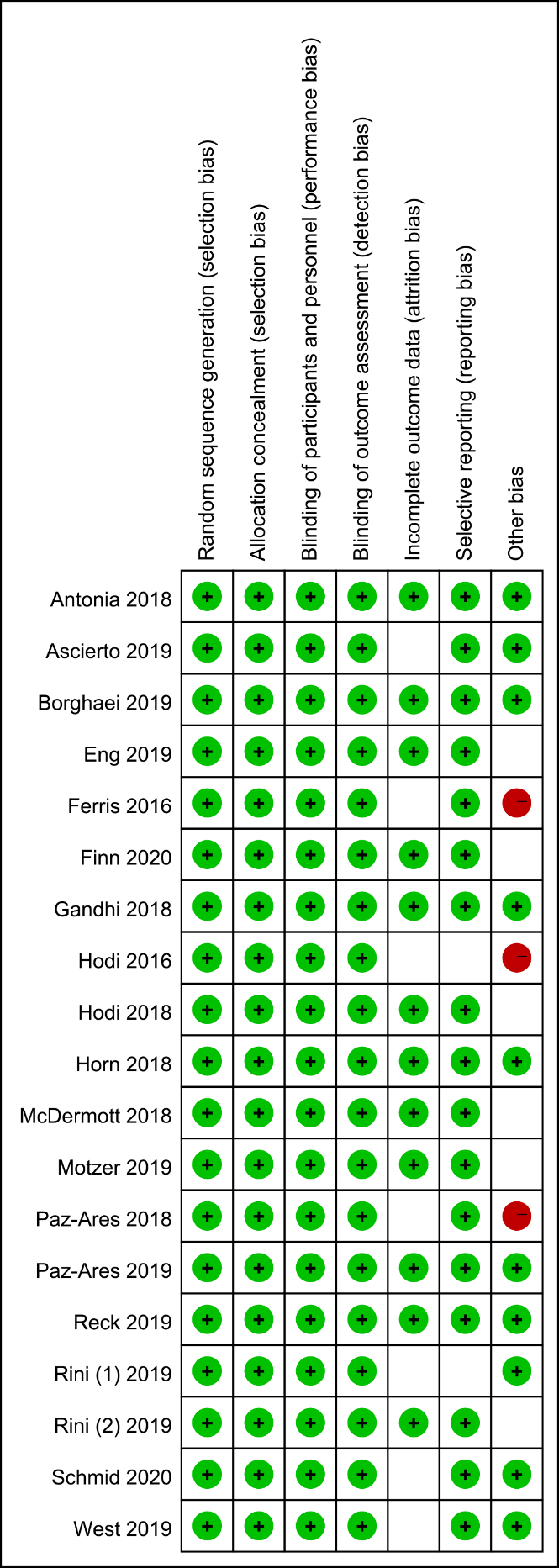


Supplemental Fig. S2 Risk of bias summary


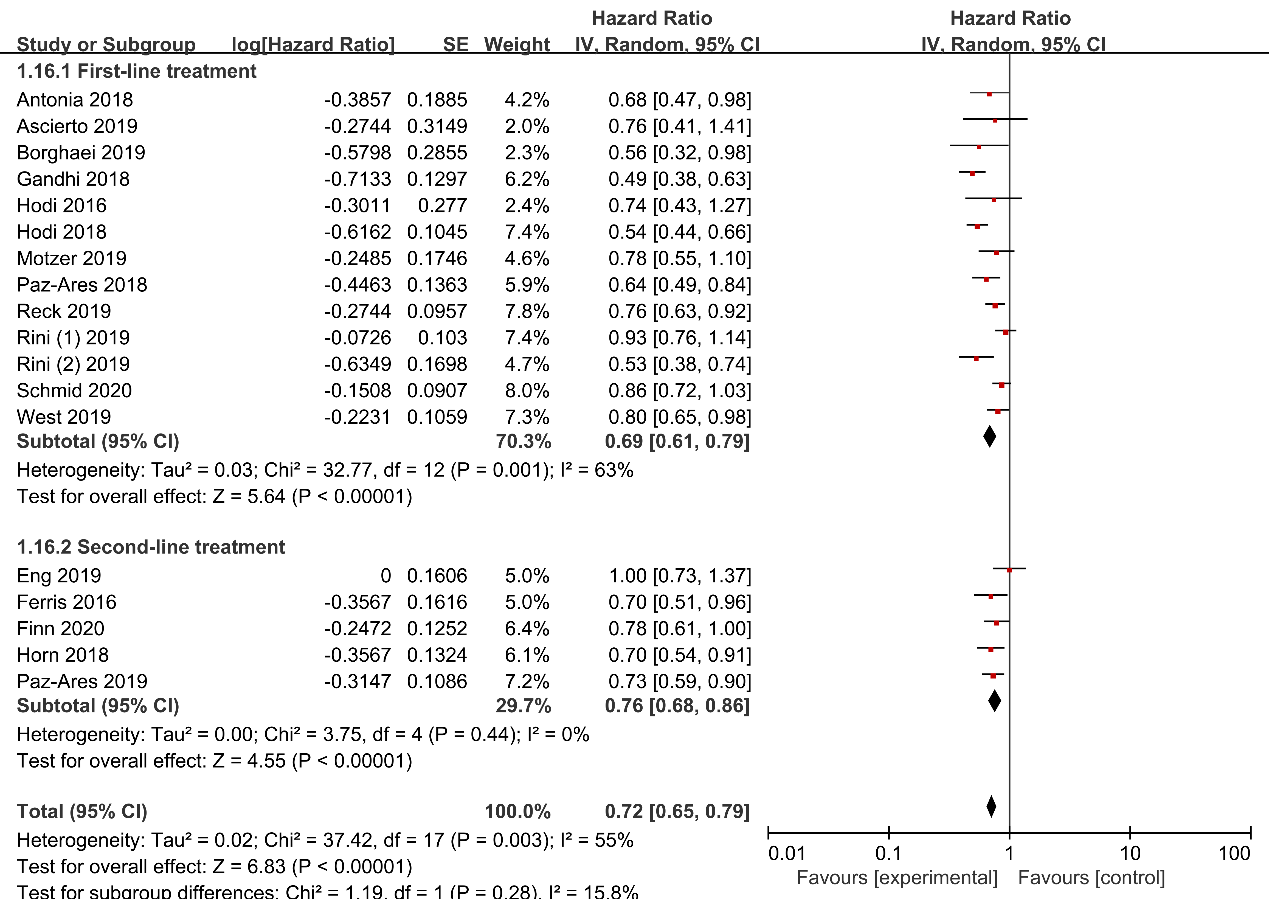


Supplemental Fig. S3 Forest plot of HR of OS based on lines of treatment


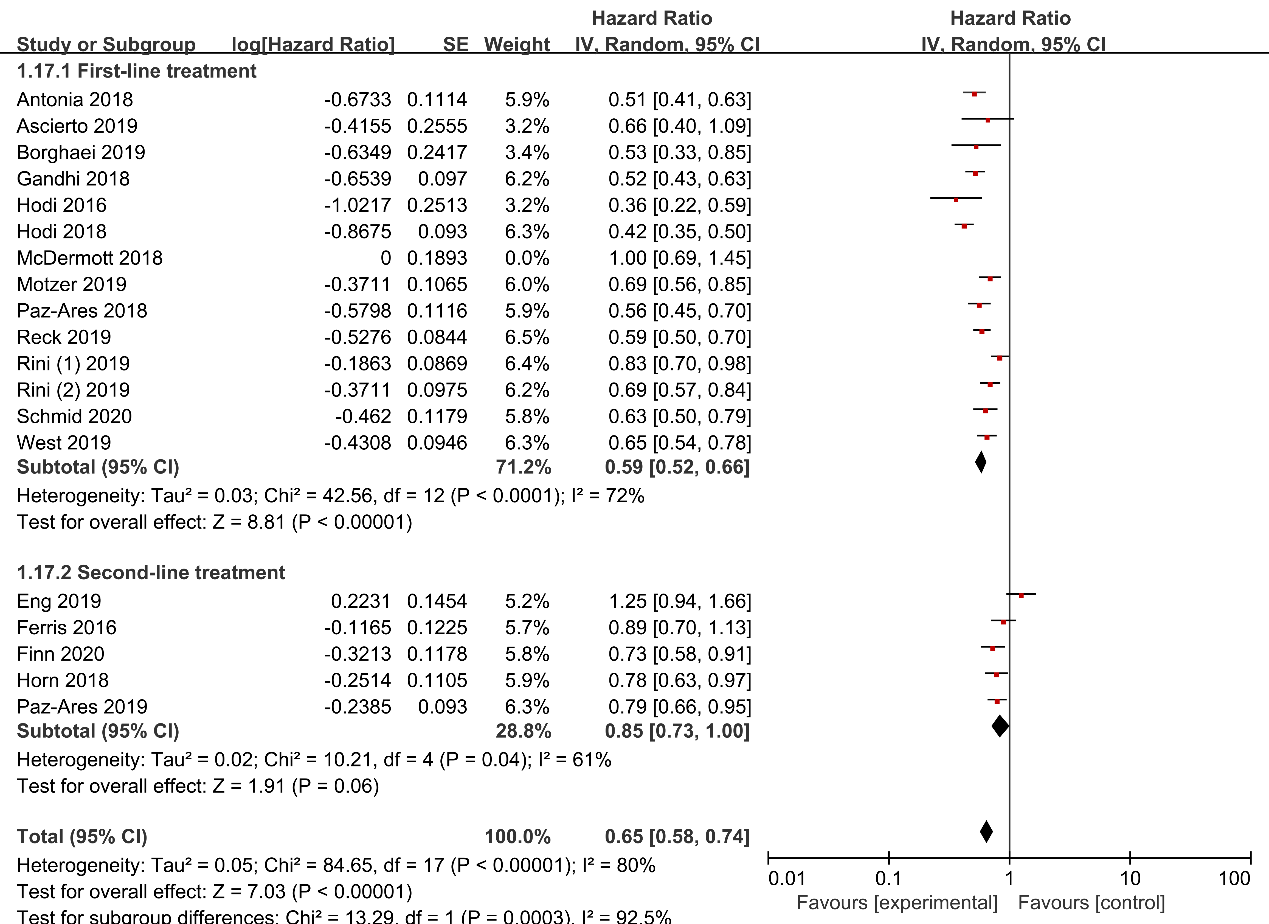


Supplemental Fig. S4 Forest plot of HR of PFS based on lines of treatment
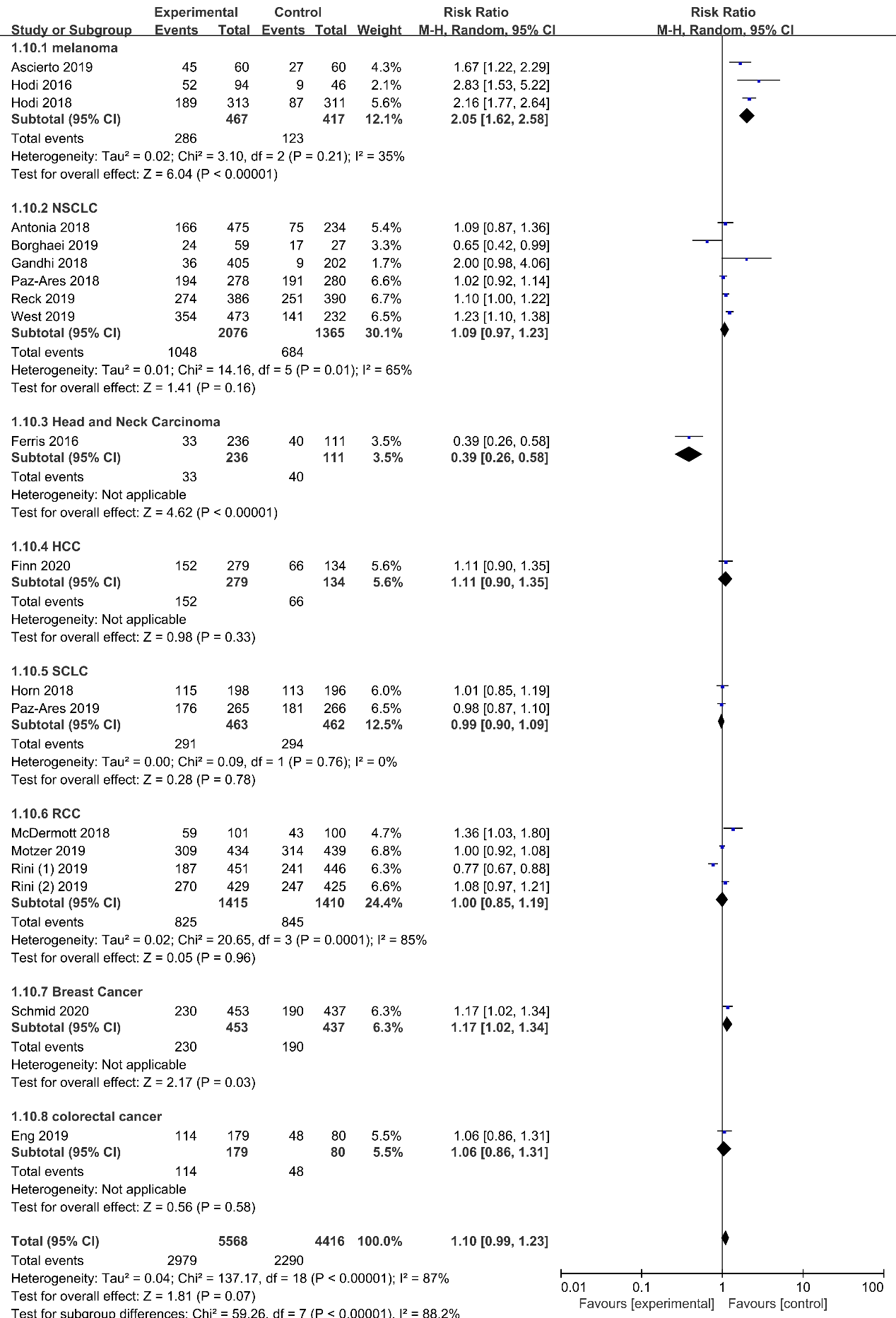


Supplemental Fig. S5 Forest plot of HR of grade 3–5 AEs rates based on tumor types


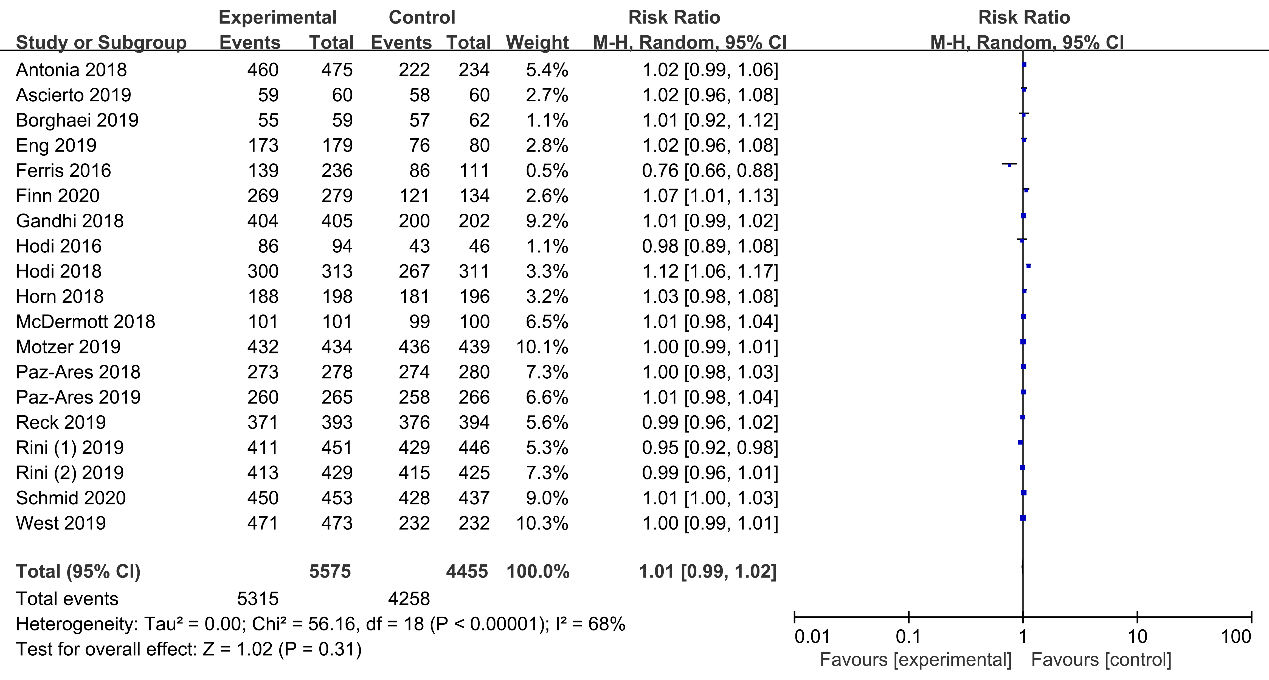


Supplemental Fig. S6 Forest plot of RR of AEs


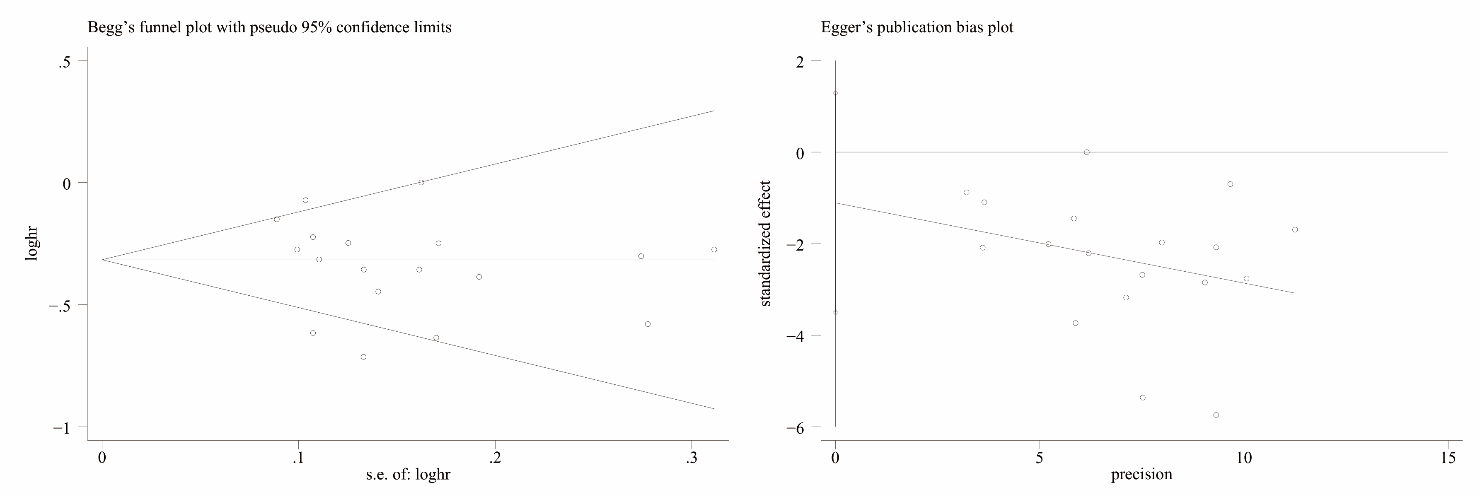


Supplemental Fig. S7 Begg’s test and Egger’s test. Begg’s test (P >0.1) and Egger’s test (P >0.1) showed no significant publication bias in OS.
